# Family Members' Experiences of Weaning From Mechanical Ventilation in Intensive Care: A Hermeneutic Phenomenological Study

**DOI:** 10.1002/nop2.70505

**Published:** 2026-03-27

**Authors:** Catarina Tingsvik, Jan Mårtensson, Fredrik Hammarskjöld, Maria Henricson

**Affiliations:** ^1^ Jönköping Academy for Improvement of Health and Welfare Jönköping University Jönköping Sweden; ^2^ Department of Anaesthesia and Intensive Care Medicine Ryhov County Hospital Jönköping Sweden; ^3^ Department of Nursing Science, School of Health and Welfare Jönköping University Jönköping Sweden; ^4^ Department of Biomedical and Clinical Sciences Linköping University Linköping Sweden; ^5^ Faculty of Caring Science, Work Life and Social Welfare University of Borås Borås Sweden

**Keywords:** family, hermeneutic phenomenology, intensive care, mechanical ventilation, nursing, ventilator weaning

## Abstract

Weaning patients from mechanical ventilation is a complex process that also affects family members, yet their perspectives remain understudied.

**Aim:**

To explore the lived experiences of family members of patients being weaned from mechanical ventilation in intensive care.

**Design:**

A qualitative, deductive design was used, inspired by the hermeneutic phenomenological research method described by van Manen.

**Methods:**

Eight family members were purposively included. The data collection consisted of personal diary notes, written during the patients' weaning phase, and individual semi‐structured interviews conducted after patient discharge from intensive care.

**Results:**

The findings were related to meaningfulness and inherent strength experienced by the family members, which were enhanced by being present at the bedside, near the patient, and involved in care. Family members shaped a temporary structure for their new everyday lives and experienced hope when thinking about the future.

**Conclusions:**

This study explores the lived experiences of family members, emphasising the importance of being near the patient, touching, maintaining contact, and communicating. Family members wish to be involved according to their preferences and capabilities. Such involvement creates meaningfulness, which further promotes family members' inherent strength. Healthcare professionals play a vital role, highlighting the advantages of adopting a person‐centred approach towards the family members by considering their resources, capabilities, and suffering, which vary over time and among different persons. To care for family members during patient weaning, healthcare professionals need to understand their needs and contributions, making the delivery of person‐centred care essential. This study highlights that person‐centred care extends beyond the patient to include family members. Recognising and supporting families as active partners in the weaning process is essential, as their involvement strengthens the well‐being of both patients and families.

**Implications for the Profession and/or Patient Care:**

Healthcare professionals should recognise family members as active partners in the weaning process and adapt person‐centred care to their individual needs and capacities.

**Patient or Public Contribution:**

Only the family members of the patients were involved in the study.

**Reporting Method:**

This study adhered to the COREQ criteria.

## 1 Introduction

Critically ill patients with failing respiratory and other vital functions may require intensive care and respiratory support, often in the form of mechanical ventilation (MV) in an intensive care unit (ICU) (Pham et al. 2017). MV is a key treatment strategy in intensive care, and patients often spend significant time on MV and during the weaning process (Goligher et al. 2016; Pham et al. 2017). As the patient's condition improves, the need for respiratory support decreases, and weaning from MV begins (Mancebo 1996). In several countries, families are allowed to visit ICUs and remain at the bedside of their critically ill relative. While the importance of family presence in the ICU is recognised, an ICU stay can be a highly stressful experience for both patients and their families (Verhaeghe et al. 2005).

## 2 Background

A patient's family is essential for safe, effective, and person‐centred care (PCC). Their support may positively affect patient experience and, to some extent, improve outcomes (Goldfarb et al. 2017; Olding et al. 2016). Moreover, the family's importance has been highlighted in research and guidelines for general healthcare (McCormack et al. 2015) and intensive care (Davidson et al. 2017; Goldfarb et al. 2020). Although family support and participation in intensive care are essential affordances for the patient, these elements remain under‐explored, particularly from the family perspective, when, for example, the patient is on mechanical ventilation (MV) and during weaning (Davidson et al. 2017; Olding et al. 2016).

The concept and process of weaning are not straightforward and are often understood in different ways. Therefore, there are significant variations in definitions, guidelines, and clinical practice for the weaning process, locally and globally (Pham et al. 2017). In general, weaning from MV is a gradual reduction of respiratory support. It is an ongoing process, covering the entire process of discontinuing the patient from MV and, most often, the artificial airway (Pham et al. 2017). Usually, weaning occurs in parallel with, the patient's improving condition; hence, weaning is a central part of being liberated from intensive care (Ely 2017). Current recommendations for liberating patients from intensive care include several aspects, for example, optimising sedation, weaning, strategies for delirium prevention, rehabilitation, and family involvement (Ely 2017; Marra et al. 2017). Awake patients have an increased need and better prerequisites to communicate and be involved in their care. Nevertheless, a patient's interaction with family, communication, and participation are complicated, due to several factors, for example, illness, patient cognition, the artificial airway, and MV (Haugdahl et al. 2017; Tingsvik et al. 2018).

Family members often experience anxiety, stress, and worry when the patients are critically ill, on MV, and need intensive care (Rückholdt et al. 2019). Previous research from the family perspective of intensive care has mainly focused on family needs and visiting policies (Leske 1991; Prachar et al. 2010) rather than providing families with opportunities to contribute to the care (Olding et al. 2016). In a Scandinavian context, healthcare professionals provide nursing and intensive care, and family members are regarded as visitors and are therefore not routinely involved in care. Their wishes and possibilities to participate relate to several factors, such as visiting policies, communicating with staff, receiving information, and being involved in decision‐making (Olding et al. 2016). However, there is a lack of understanding regarding the meaning and implementation of family participation in intensive care, as well as the impact this has on their experiences.

PCC promotes patient participation (Ekman et al. 2011); this may optimise patient experience and health outcomes in different contexts (Goldfarb et al. 2017; Kleinpell et al. 2018; Pirhonen et al. 2017). The practical uses, impacts on care, and family experiences of PCC in intensive care are less studied and present some complicating factors for PCC related to a patient's critical condition (Sevransky et al. 2017). Furthermore, limited data are available for evaluation of the weaning process from the family perspective. The published studies (Dale et al. 2020; Happ et al. 2007) have focused on prolonged MV and weaning, showing the importance of family support and encourage their presence in intensive care. Intensive care presents certain challenges for PCC related to a patient's critical condition. For example, patients on MV often have difficulty communicating, as their cognitive status is frequently affected and may vary (Sevransky et al. 2017). Adopting a person‐centred approach emphasises the importance of involving the family as a resource for the patient, particularly in bridging communication difficulties (Davidson et al. 2017; Ricœur 1994).

However, to improve care, optimise the support provided by family members, and promote PCC in intensive care during weaning, an understanding grounded in the lived experiences of family members is needed.

## 3 The Study

### 3.1 Aim

The aim of this study was to explore the lived experiences of family members of patients being weaned from MV in intensive care.

## 4 Methods

### 4.1 Design

To explore family members' lived experiences, a qualitative, deductive approach was used. The research process was guided by van Manen's (1997) hermeneutic phenomenological method and the four lifeworld existentials: lived space, lived body, lived time, and lived human relation. The study is grounded in hermeneutic phenomenology. While hermeneutic phenomenology as a philosophical movement originates in the works of Heidegger and Gadamer, van Manen has been influential in operationalising and popularising this approach within empirical human science research (van Manen 2014). His lifeworld existentials (van Manen 1997) were therefore used as an interpretive framework to guide the analysis.

The choice of a deductive approach was motivated by the research process of and findings from a study exploring patients' experiences of weaning (Tingsvik et al. 2018), which demonstrated the benefits of using the lifeworld existentials (van Manen 1997) to explore lived experiences in the intensive care context. Hermeneutic phenomenological research aims to interpret personal meanings and experiences into knowledge and thus gain a deeper understanding of a phenomenon (van Manen 1997). For this research question, it means understanding the meaning of being a family member when the patient is on mechanical ventilation and weaning.

In this paper, the word ‘patient’ is used to describe the person being cared for on MV in the intensive care unit (ICU), with the intention to clarify the text. However, it is obvious that ‘the patient’ did not occupy this role for the family members, and was instead a dear loved one. The patients were not included in the study.

### 4.2 Theoretical Framework

This study was conducted using hermeneutic phenomenology inspired by van Manen (1997), a human science studying persons and focussing on the unique and essential. One way to understand lived experiences is by identifying the fundamental existentials that go through all people's lifeworld, which form the basis of people's lives, how they experience the world, and their lived experiences. These existentials are the lived space, lived body, lived time, and lived human relation (van Manen 1997).

The existential *Lived space* describes the lifeworld from a ‘spatial’ aspect, that is, the perceived environment. The lived space appears in different ways and is crucial for a person's experiences and feelings. For example, the room we live in affects us and how we experience our lifeworld. *Lived body* ‘refers to the phenomenological fact that we are always bodily in the world’ (van Manen 1997, 103). The lived body places us in this world, moves us through life, and gains access to the world. Through the body, we experience our lifeworld. Through the body, we communicate and shape relationships with each other. *Lived time* is about the subjective experience of time (as opposed to objective time that can be measured and calculated). Time is experienced differently depending on what we do and where we are. Lived time is a person's way of being in the world where the past, present, and future come into play. *Lived human relation* intends to seek meaning and create an image of ourselves through others. ‘Lived relation to the other is the lived relation we maintain with others in the interpersonal space that we share with them’ (van Manen 1997, 104). The lived relation is an experience of others in relation to ourselves (van Manen 1997).

### 4.3 Study Setting

The study was conducted in a seven‐bed mixed ICU at a county hospital in Sweden with a nurse–patient ratio of 1:1.4. There was one room dedicated to family members for rest and two rooms for privacy and conversations. There were no restrictions for visiting hours.

### 4.4 Inclusion and Exclusion Criteria

Eligibility criteria included being a family member of a patient being weaned from MV in the ICU. Inclusion criteria were: (i) family member (age ≥ 18 years), who visited the patient regularly daily during the ICU stay; (ii) expected time for the patient on MV ≥ 3 days; and (iii) ability to speak and write Swedish.

A purposive sampling strategy was employed to capture a variety of experiences among participants, reflecting differences in age, sex, and relationship to the patient (e.g., spouse, child, parent, sibling, or friend). The sampling process was guided primarily by the aim of achieving variation rather than by numbers. Eligible participants were invited to participate in the study once the patient's condition had stabilised (i.e., within 1–4 days after ICU admission). The nurse shift leader identified potential participants, provided a brief overview of the study, and introduced them to the researcher (CT). The researcher then provided detailed written and verbal information about the study, answered questions and obtained informed consent. In total, 13 family members of 13 unique patients were invited to participate. Five declined, resulting in eight participants.

### 4.5 Data Collection

**FIGURE 1:**
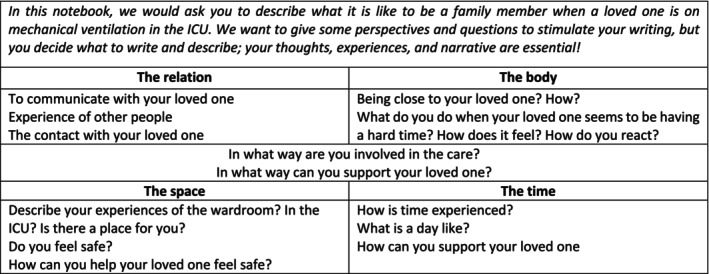
Instructions for writing the diary notes.

Data were collected between January 2019 and January 2020. Two techniques were used: personal diary notes, followed by individual semi‐structured interviews. Eight participants were asked to write diary notes based on their experiences when the patient was on MV in the ICU. The participants were given a notebook that included writing instructions (Figure 1) based on the lifeworld existentials (van Manen 1997) and included relevant aspects for the ICU context. In addition, they were asked to focus on their narrative and experience.

The semi‐structured interviews took place 6–10 weeks after ICU discharge. Two participants did not take part in the interviews (reasons were not requested) but permitted the inclusion of their diary notes in the study; thus, six interviews were conducted. Participants chose the location for the interviews, which could be either their home or the hospital. The interviews lasted between 32 and 54 min and were conducted by the first author.

**FIGURE 2:**
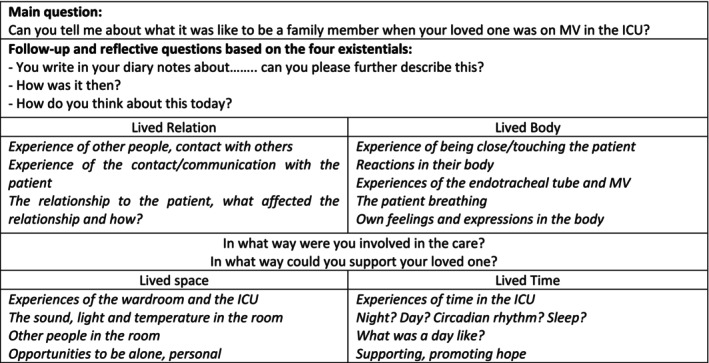
The template for the interview guide.

The diary notes were read iteratively, and text sections linked to the study aim were identified. Based on a template (Figure 2), an individually tailored interview guide was used to ensure that essential aspects were not missed, focusing on the lifeworld existentials (van Manen 1997) in conjunction with the participants' diary notes. The guide served as a support during the interviews rather than a strict questionnaire.

### 4.6 Data Analysis

The diary notes and the interviews were transcribed verbatim. The analysis started in parallel with the data collection by briefly analysing the diary notes before the interview. For further data analysis, the unit of analysis consisted of the eight diary notes, the six transcribed interviews, and research notes taken during the interviews. The analysis followed van Manen's (1997) stepwise interpretive approach rather than using a predefined analytical instrument. Several parallel and intertwined activities were conducted simultaneously, based on three approaches: holistic reading, selective reading, and detailed line‐by‐line reading (van Manen 1997). *The holistic reading* included the transcription and repeated reading of the unit of analysis to grasp the overall meaning. Throughout the analysis, the data were interpreted beyond participants' descriptions and repeatedly read to explore the essentials, answering the question, ‘What is this about?’ Interpretations were formulated to move beyond participants' accounts and to answer the question, ‘What does this reveal about the meaning of being a family member during weaning?’ *The selective reading* included identifying meaning units of the transcripts answering the question ‘What does this section reveal about the phenomenon?’ The sections were highlighted and interpreted thematically based on the four existentials, which were used as an interpretative lens to organise the meaning units. Research notes were taken to clarify the interpretation. During this, a map of the analysis and the research notes was developed. This map was constantly re‐evaluated, revised and discussed within the research team and served as the foundation for further analysis as well as the emerging themes and subsuming themes. The overall interpretation emerged by moving between the parts and the whole and searching for the underlying meaning. *The line‐by‐line reading* endorsed the emerging themes and subsuming themes by identifying several quotations to strengthen and confirm the interpretative understanding. Themes and subsuming themes were established by focusing on the experience as a whole and identifying meaningful phrases and interpretations (van Manen 1997).

### 4.7 Ethical Approval and Considerations

The study followed the principles outlined in the Declaration of Helsinki and was conducted in accordance with ICN's (International Council of Nurses 2021) code of ethics. Ethics approval was obtained from The Regional Ethical Review Board in Linköping, Sweden, no. 2018‐121‐31.

The four ethical principles of clinical research (respect for autonomy, beneficence, non‐maleficence, and justice) guided the study and were followed throughout the research process. Research involving vulnerable persons requires careful reflection and adherence to ethical principles. During the interviews, it was crucial to maintain a respectful attitude while encouraging participants to share their experiences. The researcher's familiarity with the phenomena and extensive experience in communicating with ICU patients and their families facilitated this balancing act. Data were securely stored (e.g., password‐protected, anonymised, with the code key kept in a locked cabinet accessible only to the first author) and will be preserved in accordance with institutional guidelines to ensure confidentiality and safety.

### 4.8 Rigour and Reflexivity

The Consolidated Criteria for Reporting Qualitative Research (COREQ) (Tong et al. 2007) was used to structure and facilitate the aspects of trustworthiness (Lincoln and Guba 1985) during the research process.

According to van Manen (1997), preunderstanding can never be disregarded but should be made explicit; this was achieved by reflecting and discussing it within the research team. The research team's broad experience in intensive and critical care, nursing, and qualitative and/or clinical research contributed to the credibility and dependability of the findings (Lincoln and Guba 1985). The first author has extensive experience as an intensive care nurse; considering the preunderstanding, this could have been an advantage (familiar with the research field) and a disadvantage (risk of affecting the data collection and analysis). One of the authors does not have intensive care experience; thus, a critical stance could be held and critical questions postponed. Furthermore, the deductive design strengthened the balance between interpretations and preunderstanding. Appealing to the lifeworld existentials (van Manen 1997) meant that adequate questions were raised during interviews, encouraging the participants to express their narratives. Considering the analysis, several strategies were used to enhance credibility: (i) parts of the analysis were performed separately by two researchers who discussed their findings and obtained consensus, (ii) the analysis was discussed several times in the research team, (iii) interpretations were constantly confirmed with what appeared in the data, and (iv) a critical attitude and balance of pre‐understanding was ensured.

## 5 Findings

### 5.1 Characteristics of Participants

The sample consisted of eight family members, each related to a different patient. The family members were aged between 27 and 73 years and included two daughters, one mother, four wives, and one husband. The patients were on MV in the ICU for 3–24 days for varying reasons. All patients were successfully weaned and there was no ICU or hospital mortality among the patients.

### 5.2 The Themes and Subsuming Themes

**FIGURE 3:**
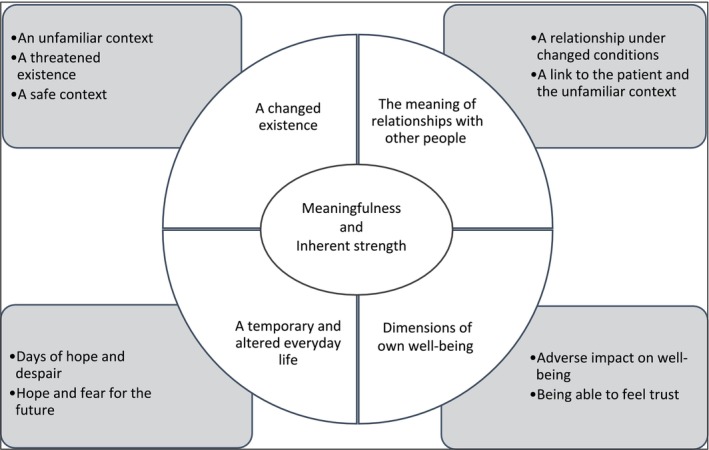
Overview of the overall interpretation (the inner circle), the themes (the circle sectors), and the subsuming themes (the boxes).

The analysis revealed four themes and nine subsuming themes (Figure 3). Common to all themes was a sense of meaningfulness and inherent strength. Being a family member during weaning was difficult and demanding, but was not devoid of hope and trust when thinking about the future. Family members had an inherent strength, and giving up or seeing things as hopeless was not considered an option. Healthcare professionals acted as a vital link between family, patient, and the new environment, supporting this sense of inherent strength. The meaningfulness and inherent strength were further strengthened by the opportunity to be involved in the patient's care. Feelings of security and daring to feel trust could be evoked, despite worries, anxiety, and fear. The family members shaped a temporary structure for their everyday lives. However, they had difficulties identifying the weaning from overall intensive care. Weaning was intertwined with the intensive care process as a whole and constituted a necessary step towards the patient's discharge from the ICU.

#### 5.2.1 A Changed Existence

When the patient was on MV in the ICU, the family members suddenly found themselves in a changed existence. The ICU context was unknown and unfamiliar and could be perceived as strange and threatening. Nevertheless, they adapted and could feel a sense of security.

##### 5.2.1.1 An Unfamiliar Context

Family members were thrown into a new context and setting. Even if some time had passed in the ICU before the weaning started, the context remained unfamiliar. The bed‐space was perceived as crowded, often with several patients in the same room. It was important not to get in the way since the staff needed space for patient care, so family members often sat on a chair close to the patient. They were also concerned about being entangled in or interfering with the medical equipment connected to the patient. Fear of accidentally affecting the equipment caused them to avoid being close to or touching the patient. The intensity of care in the room varied: sometimes it was calm and quiet, while intense work could be taking place at other times. Although family members were highly aware of the activity in the room, they perceived everything as being surreal and foreign; that is, their existence felt more like a dream than reality. To avoid unpleasant situations, they sometimes left the room, for example, when the airway was being cleaned or during extubation.

… when they were extubating, I didn't want to be there … it was almost like you felt some kind of shared pain … it hurt me as well… (N)

This was one way to adapt to the new everyday life. It was essential to support the patient as much as possible by being present in the ICU.

##### 5.2.1.2 A Threatened Existence

Although the family members felt frightened because the patient's life was at risk, and they believed that life hung by a fragile thread, they wanted to be in the ICU. The physical environment in the ICU and the altered life situation were perceived as being threatening, as this was strongly associated with life and death.

… the ICU is something you associate with life and death, I would say, somewhere in between … especially the ventilator is something you associate with that … being somewhere in between… between life and death … pretty heavy, really… (N)

The room, repleted with medical equipment and healthcare professionals, was perceived as being frightening and threatening. At the same time, these external circumstances were not seen as being of primary importance; family members understood that the patient needed these technical and medical equipment. Their focus was that the patient could stand the illness and survive.

##### 5.2.1.3 A Safe Context

Although preconception of intensive care as being unpleasant, frightening and anxiety‐inducing, their lived experience was different. This was somewhat of a paradox: they felt the greatest sense of security despite the patient being severely ill. They grew accustomed to the situation and experienced a safe context.

… the first few days and nights were bad, but then … on around the third day and after that, you got to know the people who were there … then it felt safer … I asked more and more questions … things and how everything works … I got, like, my own limited experience of it … (N)

It was of great importance that the patient was never left alone. This created a sense of security for the patient as well as for the family members. Despite the severe circumstances, healthcare professionals and family members could sometimes talk about everyday things and even laugh together. This gave a sense of relief, which furthermore increased the feeling of security. The secure context was based on the feeling that the patient was in good hands, as the staff were caring, calm, and professional.

#### 5.2.2 The Meaning of Relationships With Other People

The relationship with the patient was central, although it took courage and energy to maintain. The illness and care needs of the patient meant that the relationship between each family member and the patient was affected. The relationships with healthcare professionals and other family members served as a form of support and helped to deal with the situation.

##### 5.2.2.1 A Relationship Under Changed Conditions

MV and the severe illness changed the possibilities to maintain a normal relationship to the patient. This required mental preparation before visits and acceptance of the disrupted balance in the relationship. Family members took on the task of giving the patient hope through various gestures, small talk, and being present at the bedside. They were attentive to how the patient was doing and conveyed this to the staff or tried to support the patient in ways they managed. Examples of this were dabbing their forehead, wiping sputum, touching, or relating stories about what was happening at home.

Supporting the patient was done intuitively and taken seriously. However, they felt insufficient and wished they could do more for the patient. This generally reflects a wish to share the burden with the patient by becoming familiar with the unknown context, thereby creating a sense of security. This burden sharing often manifested as trying to continue with everyday habits and creating a relaxed atmosphere. Making small talk with the patient about everyday occurrences created a sense of security and hope. However, as communication was impaired, it was difficult and frustrating not being able to talk to one another as usual.

… I held his hand and even before we went to the hospital, I started thinking about how to talk to him … it was difficult… I just accepted the situation … it was okay to just sit quietly beside him … (H)

There was a significant sense of frustration due to the inability to understand each other and engage in communication that was meaningful. The ‘normal relationship’ with the patient was deeply missed, and the family members longed for the patient's recovery. To bridge this emotional gap, they resorted to visiting in the ICU, physically connecting with the patient, such as touching, lowering the bed rail to get closer, or holding the patient's hand.

##### 5.2.2.2 A Link to the Patient and the Unfamiliar Context

The relationship with healthcare professionals was essential; it served as a link between the family members, the patient, and the changed existence. The family members felt welcomed, seen, and actively involved in the patient's care, in the manner they wished. Their life situation became more logical, secure, calm, and filled with hope through information and conversations with healthcare professionals. The information was mostly comprehensible but sometimes overly detailed. What they valued most was complete transparency, ensuring that nothing was concealed or withheld, whether it was positive or negative news. The family members were not only passive recipients of information but participated in mutual information exchange. They asked many questions and told healthcare professionals about the patient to give them some idea of the patient as a person.

… I try to participate in care by trying to explain to the staff what I think (the patient) wants or how she is doing … (M)

The family members saw it as their duty to be present and interpret the patient's wishes.

#### 5.2.3 Dimensions of Own Well‐Being

The family members adapted their feelings and reactions to what was happening or what was expressed by the patient. Their well‐being was affected, and their reactions had two sides: on the one hand, worries, anxiety, and a sense of not being able to relax was apparent; but on the other, their well‐being was promoted by having the courage to feel trust and daring to let go of control.

##### 5.2.3.1 Adverse Impact on Well‐Being

Experiences of helplessness emerged when the patient seemed to be suffering or struggling. It was like being a spectator along a courtside and not being able to contribute to the care. It was challenging to see the patient struggle to breathe or have a severe cough. The family members felt empathy and great relief when the patient's difficulties were relieved.

… every time she had about of coughing, that was difficult … it was not natural, what happens when she coughs in it (the tube) … it just pulsated … and then I thought ‘what is going on?’…I felt (emphatically) that there was something that was irritating her airway and you want to get it out but you cannot … then the nurse sucked the tube … (N)

The well‐being of the family members was affected by the significant burden they bore. Everyday life was navigated with an energy that they did not possess, denying their exhaustion to both themselves and others. They experienced a reduced appetite and insomnia. Their memories of the ICU were tumultuous and blurred, often intensified and distorted in their dreams. The emotional burden, often leading to worry, anxiety, and fear, was substantial and present, affecting everyday life. Words such as shocking, unpleasant, fearful, and stressful were used to describe how the situations were perceived.

##### 5.2.3.2 Being Able to Feel Trust

Although family members were significantly affected by feelings of concern and anxiety, they were determined to cope with the situation. Any sign of patient recovery helped to feel trust and let go of control.

It was essential to leave the ICU on different occasions and to get a break. This increased their psychological energy and gave them the strength to carry on. Feeling that the patient was well cared for helped them to dare to let go of control and leave the ICU for a while.

… you had no control when you were not there … but there was a balance there and finding some kind of … like, letting go a little bit … taking care of yourself a little as well … (N)

However, seeing with their own eyes that the patient was doing well and that everything seemed calm and under control created trust.

#### 5.2.4 A Temporary and Altered Everyday Life

The days in the ICU were unlike anything experienced before. The days were characterised by sudden changes in positive and negative directions regarding hope, confidence, and concern for the patient. Worries about the patient and the future were intense, but family members were keen to hang on to things that could increase hope. Over time, the family members became accustomed to the situation and realised that they created new routines in their everyday life.

##### 5.2.4.1 Days of Hope and Despair

The days in the ICU were like a rollercoaster, where new information and hopes of improvement could suddenly quickly deteriorate. The patient's condition could quickly change for the better or for worse, and family members did not have time to prepare for these various. They found it difficult to keep pace with the patient's situation and the information they received from healthcare professionals. Decisions on the patient's treatment and monitoring were often made quickly, and these could be perceived as being sudden and sometimes unexpected. For example, the decision to remove the endotracheal tube or move the patient from the ICU to the ward could be made quickly. Family members considered that the patient's need for breathing support was a central matter; when the patient managed without MV for a shorter or longer time, this was a clear sign that things were moving in the right direction. Each step in the weaning process was significant and built confidence and hope.

The ICU stay led to a new, temporary everyday life with new habits. Intuitively, new day‐to‐day routines were created.

… I just wanted to be there … this became everyday life … I created a routine … I would get up in the morning and start with a walk … then I would go home, have breakfast, take a shower and go to the hospital… (A)

It became essential to have a structure for day life and a fixed schedule. Some adjusted quickly to this new life, while others had more difficulties in finding new routines.

##### 5.2.4.2 Hope and Fear for the Future

The hope that everything should turn out well helped to deal with the situation. A smile or other communicative response from the patient raised hope for the future. Family members also tried to motivate the patient to think about a positive future, even though they were concerned and uncertain on this matter themselves. Their concern and uncertainty covered everything from how the patient would manage without MV to monitoring or bedside staff around the clock, and whether the patient would ever come home again.

… it was the thought of it all … that she was there at the ICU … and what will happen next … will she come home … or not come home … … that was running through my mind all the time … (E)

As time went on, the family members adapted, found hope, and overcame their fears for the future.

## 6 Discussion

The main findings of this study were the experiences of meaningfulness and inherent strength expressed by the family members, as interpreted through the themes. Experiences of meaningfulness include being seen, involved, and needed by another person (van Manen 1997). In this study, meaningfulness and inherent strength were facilitated by the relation to the patient and the healthcare professionals, holding on to hope, feeling trust, and the experience of a safe and meaningful existence. When the patient was weaning, it was difficult, demanding, and anxiety‐inducing, but not hopeless. As previously shown in research from the ICU (McKiernan and McCarthy 2010; Rückholdt et al. 2019), the opportunity to be present in the ICU, be involved, be near, and communicate with the patient is essential for positive experiences. This study emphasises that family involvement had to be according to and in balance with family members' well‐being and capabilities, which varied between each family member and over time. According to van Manen (1997), feelings in one's body and well‐being are connected to time, relations, and contextual circumstances. When the family members experienced meaningfulness, this can be interpreted as giving rise to an inherent strength that enabled them to cope with the situation. On the other hand, if they found it difficult to keep up with the events in the ICU, they did not experience meaningfulness. Hence, their inherent strength was fragile and healthcare professionals need to promote such meaningfulness by adopting PCC for both patients and their family members.

According to van Manen (1997), lived space has a significant influence on how we experience a phenomenon and how we refer to our reality. The ICU context can be interpreted as a lived space that was perceived as unfamiliar, yet simultaneously provided a sense of security. It was essential to visit and be present in the ICU; this promoted an inherent strength and a sense of meaningfulness. According to previous research regarding family in the ICU, being near the patient is one of their most basic and essential needs (Leske 1991; Verhaeghe et al. 2005). Our study emphasises that family members' feelings are divided: they want to support the patient in various ways, but do not always have the strength and capability to do so. Healthcare professionals need to be aware of this and support in balancing their needs and capabilities (Olding et al. 2016).

Although it is well known that family members need to be present in the ICU, the possibilities for this vary significantly over the world (Goldfarb et al. 2020; Nassar Junior et al. 2018; Ning and Cope 2020). This depends on several factors, such as practical reasons, cultural expectations, and the attitudes of the healthcare professionals regarding the presence of family members (Ning and Cope 2020). This study emphasises the importance of promoting an open visiting culture in the ICU and the need for the families to feel welcome, invited, and a part of the team in the ICU. According to van Manen (1997), personal growth occurs in the context of relationships. The relationship between the family members and the healthcare professionals may serve as a bridge to reach the patient. Healthcare professionals need to take the initiative and meet the family member based on a person‐centred approach and thus guide them into the ICU context and the patient (Davidson et al. 2017; Ekman et al. 2011).

Family members are a central part of care in the ICU, and their roles can take different forms, such as a visitor, supporter, facilitator or historian (McAdam et al. 2008). Our findings illustrate that involvement in care varies between each family member and over time. Occasionally, they had the possibility and capability to take a more active part in care. However, during other times, a short visit was all they could manage or required. It is an advanced nursing task to ensure that family members are present and involved with the patient, as well as to monitor their well‐being and capabilities. Based on this knowledge, healthcare professionals need to understand and consider each person's capabilities, resources and suffering, and adopt a person‐centred approach towards the patient and family members (Ricœur 1994). This conclusion needs to be established, structured, implemented and further researched.

Being near the patient was essential, as it gave family members strength and a sense of meaningfulness. However, it could also induce frustration due to impaired communication with the patient. Therefore, encouraging and supporting the relationship between family members and the patient is essential to create hope and meaning for both parties (Davidson et al. 2017; Tingsvik et al. 2018). According to previous research (Egerod et al. 2015; Rückholdt et al. 2019), being near and having contact with the patient is a key factor for family members to allow them to cope with the situation, which our study emphasises. By being near the patient, the relationship is maintained, and they find meaning in being in the ICU, which brings hope for the future that they may transfer to the patient (Valle and Lohne 2021).

From the perspective of lived time, the findings illustrate how hope was sustained by family members through an orientation towards the future (van Manen 1997). Hope was essential, both for family members and to instill this feeling in the patient. Family members had a difficult time understanding the weaning process, which was intertwined with the intensive care process; this has previously been described by patients (Tingsvik et al. 2018). Occasionally, they found it difficult to keep up with the events in the ICU when deterioration or medical treatment could appear quickly and unexpectedly. Their lived experience was described as they lost their balance, were pulled out of the safe context, and experienced new uncertainty. To control this, healthcare professionals have to consider the importance of family members to keep pace (Frivold et al. 2015). Being involved in care may be one way to optimise the family members' understanding of the intensive care and the patient's critical condition and recovery (Frivold et al. 2018; Valle and Lohne 2021). This approach might help to prevent emotional stress during the rollercoaster between hope and negative feelings and support their experiences of meaningfulness and inherent strength. Further research is needed regarding involving family members in the MV weaning process in the ICU.

### 6.1 Strengths and Limitations of the Work

The purposive sample was limited, due to its small size and a predominance of female participants, which may restrict the transferability of the findings. Moreover, the participants comprised family members who visited the ICU, potentially introducing a selection bias. The intention was to include several men, but the COVID‐19 pandemic disrupted the data collection. However, data collection generated rich material and therefore strengthened the overall trustworthiness.

### 6.2 Recommendations for Further Research

Further research is needed to explore how family members can be better supported during weaning and how their involvement in care can be optimised. Healthcare professionals should adopt a person‐centred approach towards the family members, which must be implemented and further studied.

### 6.3 Implications for Policy and Practice

A person‐centred approach helps healthcare professionals to involve family members in care, especially when patients are weaning from MV. It allows them to tailor their involvement to match their capabilities, resources, and experiences of suffering. Furthermore, a person‐centred approach is likely to enhance the resources of family members and reduce their suffering.

## 7 Conclusion

The findings indicate that family members' experiences of meaningfulness and inherent strength emerged through being near the patient, maintaining contact, and being involved in care during the weaning process. Being a family member to a patient in the ICU was difficult, demanding, and anxiety‐inducing; however, several aspects helped family members find meaning and hope. The opportunity to be near, touch, have contact, and communicate with the patient promoted the experience of a safe and confident context and was essential for meaningfulness.

Family members desire to be actively involved based on their preferences and abilities. As a result, healthcare professionals should guide and support family members in the ICU, as such involvement creates a sense of meaning and further enhances their inherent strength. This highlights the importance of adopting a person‐centred approach towards family members by considering their resources, capabilities, and suffering, which vary over time and among different persons.

## Author Contributions

**Catarina Tingsvik:** conceptualisation, methodology, validation, investigation, formal analysis, writing original draft, writing review and editing. **Jan Mårtensson:** conceptualisation, validation, writing review and editing. **Fredrik Hammarskjöld:** conceptualisation, validation, writing review and editing. **Maria Henricson:** conceptualisation, methodology, validation, formal analysis, writing review and editing, supervision.

## Funding

The study was supported by grants from Futurum—the Academy for Health Care, Region Jönköping County, Sweden.

## Ethics Statement

This study was performed in accordance with the Declaration of Helsinki and its revisions. Ethical approval was obtained from the regional ethical review board of Linköping, Sweden (Dnr 2018/121‐31).

## Consent

Informed consent was obtained from all the participants. The study obtained ethical approval from The Regional Research Ethics Review Board in Linköping, Sweden, no. 2018‐121‐31.

## Conflicts of Interest

The authors declare no conflicts of interest.

## Data Availability

Data is not available to the public due to privacy and ethical restrictions.
